# The Dynamic Roles of Mesenchymal Stem Cells in Colon Cancer

**DOI:** 10.1155/2018/7628763

**Published:** 2018-11-07

**Authors:** Shan Wang, Zhiguo Miao, Qiyuan Yang, Yimin Wang, Jinzhou Zhang

**Affiliations:** ^1^College of Animal Science and Veterinary Medicine, Henan institute of Science and Technology, Xinxiang, Henan, 453003, China; ^2^Department of Molecular, Cell and Cancer Biology, University of Massachusetts Medical School, Worcester, MA 01605, USA

## Abstract

Colon cancer is still one of the most common causes of cancer in human and is characterized by lymphocyte infiltrates and originates from the epithelial cells found in the lining of colon or rectum of the gastrointestinal tract. Mesenchymal stem cells (MSCs) are composed of the multipotent stem cell group of stroma and can be differentiated as various cell lineages, such as fibroblasts, osteoblasts, and adipocytes. MSCs provide mechanical and structural support and have potential functions during tumor growth and metastasis. The efficacy of MSC-based therapies is partly dependent on the migration and homing of MSCs to tumors and metastatic sites. However, their migratory and engraftment potential is poorly understood. In this review, the characteristics and mechanisms of MSC's dynamic interaction with colon cancer were summarized, particularly the potential functions of MSCs on colon cancer, including its role in improving tumor growth and as a potential candidate for tumor therapy. Understanding MSC homing provides new insights into the manipulation of MSC and the improvement of their efficacy for colon cancer therapy.

## 1. Introduction

Colorectal cancer (CRC) is one of the most common cancers diagnosed in humans and is a cause of mortality in worldwide [1]. CRC is a complex disease that begins with adenoma, which occurs through complicated process and reasons. It was reported that accumulation of mutations in epithelial and preneoplastic cells are probably major reasons [2]. The causes of CRC are thought to be linked to a combination of life habits and genetic factors, such as smoking, aging, diet, and obesity [3]. In many cases, colon cancer is characterized by lymphocyte infiltrates, and the majority of deaths due to CRC are caused by therapy refractory metastasis [4]. Transformation of tumor cells and tumor formation are promoted by factors, including cellular and noncellular components known as the tumor stroma and tumor environment [5]. The “seed and soil” theory proposed in 1889 by Stephen Paget explained that a cancer cell (seed) would only proliferate when the environment (soil) is suitable [6]. Thus, the microenvironment is essential for tumor growth.

The unique microenvironment of colon cancer is composed of many types of cells, such as fibroblasts, immune cells, and vascular cells [7]. All these cells contribute to the survival and growth of the tumor. Fibroblasts in the stroma of various human carcinomas are the important type of cell resource considered to contribute cancer metastasis and growth, which can also suppress the anti-tumor immune response [8–10]. The tumor microenvironment on the colon cancer cell invasion, metastasis, and resistance against drugs involves the communication of fibroblasts with cancer cells directly or indirectly [11, 12]. Especially in colorectal cancer, direct contact between cancer cells and MSC is rather than indirect contact [13]. Therefore, these cells appear to decrease the survival prospects of patients. Mesenchymal stem cells (MSCs) are one of major cellular constituents in intestinal tumor, which contribute to carcinogenesis via interaction with other cell types in the tumor environment [14]. As described by studies, the activities of MSCs during colon cancer help increase the understanding of their functions [15, 16]. Mounting evidence shows the suppression function of microenvironment in tumor growth* in vitro*, such as Hela cells (human cervical cancer line), HT29 cells (human intestinal epithelial cell line), SW480 (human colorectal adenocarcinoma cell line), Lovo cells (human colorectal adenocarcinoma cell line), and HCT-116 cells (human colorectal carcinoma cell line) [17–22]. Both the proportion and the localization of stromal cells in microenvironments indicate that the function of these cells is critical to tumor growth and metastasis [23, 24].

To further explore the role of MSCs in colon cancer development, in this study, the characteristics and mechanisms of MSC interaction with colon cancer were reviewed, with focus on the impact of MSCs on colon cancer and how MSCs act as vehicle for tumor treatment.

## 2. Characteristics of MSCs

MSCs are nonhematopoietic precursor stem cells with multiple abilities to differentiate into several types, such as adipocytes, fibroblasts, osteocytes, and neurocytes [25]. MSCs are originally derived from mesodermal progenitors and are present in bone marrow, adipose tissue, umbilical cord, and muscles [26, 27]. The hallmark of human MSCs is its common phenotype expresses CD44, CD73, CD90, CD105, stromal cell antigen-1, and leukocyte function-associated antigen-3 and has the following receptors on its surface: interleukin (II) receptor, *γ*-interferon receptor, and tumor necrosis factor *β* (TNF*β*) receptor, but not FasL, MHC-II [27, 28]. MSCs can be isolated from different tissues, such as bone-marrow (BM), adipose tissue (AD), amniotic fluid, and umbilical cord blood (UCB) [29].

Currently, BM-MSCs, AD-MSCs, and UCB-MSCs are mainly applied for cancer therapy. Bone-marrow-derived MSCs are the most commonly used and also the earliest described MSCs [30]. However, application of BM-MSCs is limited by their low migration compared with other MSCs and complex extraction methods [19]. AD-MSCs are extracted from fat tissues, which are easily obtained, and once isolated have a similar biological morphology and function that make them appealing candidates for stem cell transplant and cancer therapy [31, 32]. USB-MSCs are a group of cells isolated from the umbilical cord blood of the newborn, which have a much stronger capacity of proliferation and continuous culture for more than 80 population doubling [33, 34]. The utilities of MSCs posed a promising way in disease therapies due to their properties. They are widely different from many adult tissues and are easy to apply. They have a homing ability and they avoid immune rejection. One of their key properties is target migration. Accompanied by tissue damage, such as inflammation, injury, or tumor, MSCs migrate to inflammation sites* in vivo*. The process of passing vascular endothelial and basilemma are dominated by the release of specific endocrinal signals from the tissues [35, 36]. The tumor tropism capacity of MSCs could be utilized to deliver antitumor biological agents effectively and avoid the toxic activity caused by the increase in high blood concentration [37]. Another feature of MSCs is their low immunogenicity because of the lack and low levels of expression of costimulatory molecules proteins, such as major histocompatibility complex class I (MHC class I) protein, MHC class II proteins, CD80, and CD86 [38, 39].

## 3. Homing of MSCs

Homing refers to the phenomenon wherein cells migrate to the organs through blood circulation or even after long migration. Studies have reported that MSCs preferentially migrate to tumor sites [40–42]. The efficacy of MSCs mainly depends on their ability to produce cytokines, and the homing of MSCs to tissue requires the secretion of these factors [43, 44]. A portion of MSC-derived fibroblast premixed with A375SM melanomas cells was observed to migrate to the tumor architecture and differentiate into fibrous capsules [40]. The homing of MSCs is regulated by various factors, such as age, dosage, and passage of MSCs. The efficiency of MSCs is attenuated by higher passages and the freshly isolated MSCs had a better homing capacity [45]. In addition, the efficiency of MSCs was decreased undergoing a long culture time, which demonstrated that the decreased potency of stem cells is due to ageing. MSCs could be derived from different tissues with differences in the multiple type of cells isolated [46]. These differences are in part due to the microenvironment from where they were isolated [47]. Aside from the source of MSCs, the culture conditions influence their characteristics. Matrix metalloproteases (MMPs), which are known to be crucial to the migration of cells, have been shown to have an important role in migration. Also, inflammatory cytokines TNF-*α* and TGF-*β*1 can enhance migration of MSCs via mediation of MMPs [48, 49].

The homing mechanism of MSCs still keeps elusive. However, various studies have shown the potential important role of receptors and adhesion molecules in homing and migration of MSCs [50–52]. Homing is partly dependent on the chemokine receptors in tumors while it acts as ligands for receptors expressed by MSCs and CXCR4 and its partner was featured in human stem cell homing [53, 54]. Apart from CXCR4, isolated MSCs also expressed CCR1, CCR4, CCR7, CCR10, CXCR5, and CXCR6 [55]. Expression of CXCL12 and CXCl2 was increased when rat MSCs were exposed to a C85 human colorectal cancer cell conditioned medium [56]. These suggest that chemokines expressed by MSCs might contribute to the tumor-homing process. Cytokines secreted by inflammatory as well as tumor, including vascular endothelial growth factor (VEGF), transforming growth factor (TGF), neurotrophic factor (NTF), FGFs, CCL2, and CXCL8 might play a role in the homing of MSCs [55, 57].

Migration and homing of MSCs require cells to attach to the endothelial cells (ECs) and enter the tumor tissue. A number of adhesion molecules, such as integrins, involved in the process of MSCs, interact with ECs [58], while blocking or knocking out integrins could help in understanding the role of adhesion molecules in homing of MSCs. Researchers found that VLA-4/VCAM-1 is needed for the adhesion of MSCs on ECs, while the binding of MSCs can be suppressed during VLA-4/VCAM-1 preincubation with antibodies [15].

## 4. Effect of MSCs on Colon Cancers

### 4.1. MSCs Enhance Growth and Metastasis of Colon Cancer

The tumor microenvironment is important for the promotion of tumor development. It is reflected by the interaction of tumor cells and stroma cells adjoining, and is mainly composed by inflammatory cells and fibroblasts [59, 60]. Studies show that MSCs may be the direct cellular target of the alterations, which leads to tumor formation, which is an important part of the maintenance of tumor microenvironment [61, 62]. After migration to the tumor microenvironment, MSCs differentiate into tumor-associated fibroblast (TAF)-like cells, which are a predominate tumor-promoting stromal cells that affect the survival and growth of colon cancer [63]. These cells exhibit functional properties and promote tumor cell growth in vitro or in vivo, as well as the expression of myofibroblast markers (*α*-smooth muscle actin and fibroblast surface protein) [64–66]. MSCs have been reported to exhibit the characteristics of TAFs reduced by colon cancer cells, and accompanied by high expression of *α*-smooth muscle actin is the transformation of MSCs to TAFs mediated by Notch signaling through TGF-*β*/Smad signaling pathway [20]. In addition, research has shown that incorporation of KM12SM colon cancer cell and MSCs with existing carcinoma-associated fibroblast (CAF) phenotype, such as platelet-derived growth factor receptor-*β* expression, caused enhanced colon cancer cell growth and metastasis [67]. Collectively, one of the mechanisms of MSCs that promotes colon cancer cell proliferation is associated with transforming into myofibroblast in tumor microenvironment.

Apart from their effect on tumor microenvironment, MSCs have also been shown to modulate colon cancer cell growth via other mechanisms. MSCs in colon cancer have been shown to promote three prospects of tumor, including angiogenesis or vascular cell formation, tissue invasion and metastasis, and suppression of apoptosis [18, 22, 67–70]. BM-MSCs pretreated with inflammatory cytokines IFN-*α* and TNF-*α* can induce VEGF expression secreted by colon cancer cells, and result in the promotion of angiogenesis in vitro [68]. The transplantation of MSCs and KM12SM caused a significantly increased number of tumors and decreased the survival rate of nude mice by enhancing migration and invasion [67]. Hogan et al. [70] found that a higher presence of MSC-secreted PAI-1 level significantly increased the migration of HT-29 colon cancer cells. SW480 colon cancer cells mixed with MSCs transplanted showed elevated capability of proliferation, rich angiogenesis, and highly metastatic ability in tumor tissues in vivo [71]. AM-MSC-produced FGF10, VEGFC, and matrix metalloproteinases (MMPs) increased the capacity of invasion and formation of colon cancer cells in vitro through the activation of Wnt signaling pathway and subsequently increased the tumorigenicity of cancer cells in murine model in vivo [68]. These demonstrate that MSCs could enhance growth and metastasis of colon cancer via various mechanisms.

Accumulating studies have shown results that MSCs promote colon cancer development. While this may the case, the effect of MSCs on tumors is still controversial. As noted in the review of Hogan et al. [70], HT-29 colon cancer cells were observed to have increased proliferation, while the cell line HCT-116 showed decreased growth. The result suggests the dual role of MSCs in colonic tumor. The majority of tumor models used in the previous studies are performed by coadministering in an immune-deficient mouse. While such models have advantages, the defection of autologous nature and aspects of chronic inflammation induced tumor is absent.

In the correlation between inflammation and colon cancer pathogenesis, it was noted that chronic inflammation is considered to play a crucial role in colorectal cancer progression [72]. A clinical study indicated that colon cancer also tends to develop in patients with inflammatory bowel disease [73]. Interestingly, the tumor microenvironment is mainly composed of inflammatory cells and is a necessary participant in the tumor progression [74]. In contrast with the promotion of MSCs on colon cancer, studies have reported the inhibition of MSCs in inflammation [75–78]. IL-1*β*-primed MSCs can improve inflammatory disorders on dextran sulfate sodium induced colitis [75]. He et al. (2012) also proved that allogenic BM-MSCs exert therapeutic effect on dextran sulfate sodium induced colitis in experimental mouse model [77].

### 4.2. MSC as Vehicles for Colon Cancer Therapy

In view of the tumor migration properties of MSCs, they are modified to deliver specific anticancer agents to tumor sites [79–81]. Modified MSCs are attracted to tumor stroma and, thus, they can be used to target deliver anti-cancer agents to multiple sites. Decreased MSC homing to tumor might suppress their effect on tumor growth. In vivo studies have repeatedly shown that MSCs are trapped after intravenous injection [82, 83]. Many existing methods are used to modify MSCs and the most commonly used are genetically modified MSCs [84, 85]. The cells can transport the agents into the tumor microenvironment and greatly reduce the immune reaction in vivo through high concentration anticancer agents target tumor site. Using MSCs as delivery vehicle, many researchers are looking into using their tumor tropic properties to inhibit tumor development [86–92]. Transfected cytosine deaminase into AT-MSCs in combination with 5-fluorocytosine can significantly enhance cytotoxicity on target CT-29 tumor cells in vitro. In vivo, CD-AT-MSCs administered in mice treated with 5-fluorocytosine caused inhibition of tumor growth and, thus, CD-AT-MSCs have the ability to deliver the CD genes to the tumor site and exert anti-tumor effect [92]. In colon cancer cell lines HCT-15 and DLD-1, it was found that TNF-related apoptosis inducing ligand (TRAIL) transgenic human MSC suppressed colon cancer growth by apoptosis induction [86]. Although TRAIL-resistant tumor cells exist, Mueller et al., 2011 [87], proved that, in selected colon cancer cells, TRAIL-MSC can overcome resistance via direct intercellular interaction, thereby inhibiting the growth of HCT-8 and HT29 cells. Moreover, the mechanisms of engineered MSCs on tumor inhibition could be partly due to the downregulated expression of VEGF [88]. Transfected MSCs with sodium iodide symporter and CCL5/RANTES treated mice showed a significant delay in tumor growth after intrasplenic injection of the colon cancer cell line LS174t [90]. Different approaches have been applied for tumor treatment, and with the depth of our understanding of MSCs, more kinds of MSC vehicles would be used for colon cancer therapy. Suicide gene transduced MSCs successfully decreased Rluc activity in CT26/Rluc cells after prodrug ganciclovir treatment [91]. Different tissue-derived MSCs may have various roles on the development of colon cancer and may be also influenced by experimental conditions in vivo or in vitro.

Recently, studies concerning MSC-derived extracellular vesicles in the treatment of tumor growth have attracted a lot of attentions. A large portion effect of MSCs is contributed to their secretion products, such as exosomes [93]. After stimulation, exosomes act in microenvironment and cellular communication, which is closely related to tumor formation. In breast cancer cells, exosomes could suppress angiogensis via downregulation of VEGF expression [94]. Meanwhile, miRNAs transferred by exosomal may promote the dormancy of the metastatic in breast cancer cells [95, 96]. In addition, TRAIL-expression MSC induced apoptosis in 11 cancer cell lines and had no cytotoxicity in human bronchial epithelial cells [97]. Collectively, the anticancer activity of MSC-derived extracellular vesicles might be potential used in tumor therapy. However, limited studies have been investigated in colon cancer cells, and more work still needs to be done for tumor therapy.

The biological effect of MSC on colon cancer is the result of multiple factors. The source of MSCs is one of important factor. BM-MSCs and AD-MSCs could enhance colon cancer growth and metastatic capacity, while UCB-MSCs are less reported. Another element is the cell type used in vitro. The effect of MSC secreted PAI-1 on colon cancer cell growth was cell-line dependent [70]. In addition, the process of colon carcinogenesis also affects the activity of MSCs. The anti-inflammatory properties of MSCs make them a tool for treating colitis-associated colorectal cancer.

MSC could promote colon cancer growth or inhibit colitis-associated colorectal cancer, and be taken as vehicle for antitumor agent delivery. One should be careful when using MSC for drug delivery to patients with malignancies, as some instances of MSC migration to tumor are not very efficient because the MSC cannot reach its target site [98]. However, this problem can be addressed by replacing the paracrine signaling from MSC that affects the tumor [99]. Instead of reducing tumor growth, engineered MSC also can cause undesired effects; IL-6 secreted by MSCs was shown to promote breast cancer cell proliferation and migration [100]. Finally, in vitro and in vivo work also have difference [101]. Further research is still needed to reveal the role of MSC and apply MSC therapy to patients in the clinical setting.

## 5. Perspectives

Controversies exist in the use of MSCs in colon cancer treatment. Research on MSC homing and migration could benefit the MSC-based tumor therapy. What appears to be an aspect of the target to tumor might have a significant impact on the efficacy of anti-tumor agents. MSCs engineered for tumor therapy is advanced, especially in colon cancer [102, 103]. Many problems on artificial vehicles can be addressed, such as immune rejection, drug accumulation in non-target tissues, and poor permeability [104, 105]. MSCs not only provide a non-immunogenicity delivery system, but also form a platform for anti-tumor drug delivery [106]. Therefore, targeting treatment with MSCs as a cell carrier is an excellent approach for colon cancer treatment. Even the biological characteristics of MSCs are not be influenced after transfection with adenovirus, retrovirus, and lentivirus [107, 108]. Gene therapy targeting the tumor based on MSCs can have an improved therapeutic effect due to the migration of MSC target to the primary and metastatic tumor sites, and activation as a mini pump, which continuously pumps various anti-tumor agents.

Colon cancer is a complex tumor and a significant advancement in MSC therapy is still limited to the metastatic disease. At present, engineered MSCs have been applied for colon cancer treatment [109]. However, the effects of this treatment have not been validated in vivo in humans. Before they can be used in the clinic, the manufacturing process with the most promising evidence should be validated. Moreover, MSCs are widely studied and applied in many clinical trials in various clinical fields, but the exact mechanism underlying the interaction of MSCs and tumor is little known. Further works would be helpful for a better understanding of MSC biology. With the development of cytology, gene therapy, and stem cell engineering, a simple and easy-work process should be developed, which might lead to widespread use of MSCs in efficacious tumor treatment.
